# Geographically Distinct and Domain-Specific Sequence Variations in the Alleles of Rice Blast Resistance Gene *Pib*

**DOI:** 10.3389/fpls.2016.00915

**Published:** 2016-06-23

**Authors:** Kumar Vasudevan, Casiana M. Vera Cruz, Wilhelm Gruissem, Navreet K. Bhullar

**Affiliations:** ^1^Plant Biotechnology, Department of Biology ETH Zurich, Switzerland; ^2^International Rice Research Institute Los Banos, Philippines

**Keywords:** rice blast resistance, *M. oryzae*, Pib, allele mining, genetic diversity, SNP, NB-ARC, LRR

## Abstract

Rice blast is caused by *Magnaporthe oryzae*, which is the most destructive fungal pathogen affecting rice growing regions worldwide. The rice blast resistance gene *Pib* confers broad-spectrum resistance against Southeast Asian *M. oryzae* races. We investigated the allelic diversity of *Pib* in rice germplasm originating from 12 major rice growing countries. Twenty-five new *Pib* alleles were identified that have unique single nucleotide polymorphisms (SNPs), insertions and/or deletions, in addition to the polymorphic nucleotides that are shared between the different alleles. These partially or completely shared polymorphic nucleotides indicate frequent sequence exchange events between the *Pib* alleles. In some of the new *Pib* alleles, nucleotide diversity is high in the LRR domain, whereas, in others it is distributed among the NB-ARC and LRR domains. Most of the polymorphic amino acids in LRR and NB-ARC2 domains are predicted as solvent-exposed. Several of the alleles and the unique SNPs are country specific, suggesting a diversifying selection of alleles in various geographical locations in response to the locally prevalent *M. oryzae* population. Together, the new *Pib* alleles are an important genetic resource for rice blast resistance breeding programs and provide new information on rice-*M. oryzae* interactions at the molecular level.

## Introduction

Rice is a staple food for more than half of the world population. Several biotic and abiotic stresses constantly threaten global rice production. Rice blast caused by the fungal pathogen *Magnaporthe* *oryzae* is the most destructive rice disease that can damage the whole plant (Wang et al., 2014), including the root (Sesma and Osbourn, 2004). It is one of the most widespread rice diseases found in over 85 rice growing countries (Spence et al., 2014). Recently, *M. oryzae* has also established itself in wheat agro-ecological systems in Argentina, which is alarming as the blast disease is becoming a significant threat to wheat production as well (Perelló et al., 2015).

Utilization of host resistance (*R*) genes is the most efficient environment-friendly and economically sustainable approach to control rice blast. To date about 100 major *R* genes and over 350 quantitative trait loci (QTLs) have been identified for rice blast resistance. Twenty-one of the *R* genes have been cloned and characterized (Liu et al., 2013; Zhai et al., 2014), including *Pi64* that confers resistance to both leaf and neck blast (Ma et al., 2015). Most of the blast resistance genes belong to the coiled-coil nucleotide binding site leucine-rich repeats (CC-NBS-LRR) class of *R* genes. The LRR domain is reported to determine the resistance specificities of various plant R proteins, whereas, the NBS domain functions as a molecular-switch in regulating the active/inactive states of an R protein (McHale et al., 2006; Tameling et al., 2006; Williams et al., 2011). There are also exceptions to NBS-LRR type, such as *Pid2* that encodes a receptor-like kinase and the recessive gene *pi21*, which encodes a proline-rich protein (Chen et al., 2006; Fukuoka et al., 2009). Some of the blast *R* genes such as *Pikm1-TS/Pikm2-TS*, *Pi5-1/Pi5-2*, *Pikp-1/Pikp-2*, *Pikh-1/Pikh-2*, and *RGA4/RGA5* are present adjacently and both genes are essential for functional resistance (Ashikawa et al., 2008; Lee et al., 2009; Okuyama et al., 2011; Yuan et al., 2011; Césari et al., 2014; Zhai et al., 2014). Despite the large number of available rice blast resistance genes, the rapidly evolving *M. oryzae* can frequently overcome resistance. Because of the severity of the disease and the role as an interesting model organism, *M. oryzae* was the first fungal pathogen whose genome was sequenced (Dean et al., 2005). To better understand the frequent breakdown of resistance, the genomes of two additional *M. oryzae* field isolates were also re-sequenced. The *M. oryzae* genomes revealed 100s of isolate-specific genes and gene duplication events. Thousands of loci contain transposon-like elements and about 200 genes were disrupted by transposable elements in all the three sequenced *M. oryzae* strains (Xue et al., 2012). Nine of more than 40 characterized *M. oryzae Avr* genes have been cloned so far (Zhang et al., 2015). Most of these *Avr* genes and their allelic variants exhibit presence/absence of transposon element (TE) insertion, frequent presence/absence polymorphisms and high rate of shared non-synonymous substitutions among different *M. oryzae* strains (Huang et al., 2014; Zhang et al., 2015). Together, such high incidence of transposon-mediated inactivation of genes involved in host-specificity, the high rates of non-synonymous mutations as well as frequent gain and loss of avirulence (*Avr*) genes can explain the dynamic nature of *M. oryzae* to overcome host resistance (Dean et al., 2005; Yoshida et al., 2009; Xue et al., 2012).

Considering the rapid evolution of *M. oryzae*, it is critically important to broaden and diversify rice blast resistance sources by identifying novel resistance genes and allelic variants of the known resistance genes. Seed banks represent a valuable resource for exploring the genetic diversity present in rice cultivars, landraces and wild relatives. Advancements in molecular marker and DNA sequencing technologies have greatly accelerated the identification of allelic variants of *R* genes from genetically diverse accessions (Kilian and Graner, 2012). Allele mining in various crop germplasm collections has identified several functional allelic forms of *Mla* (powdery mildew resistance gene in barley), *L* (rust resistance gene in flax), *Pm3* (powdery mildew resistance gene in wheat) and *Pi54* (rice blast resistance gene) genes that confer race-specific or broad spectrum resistance (Ellis et al., 1999; Bhullar et al., 2009; Seeholzer et al., 2010; Thakur et al., 2015; Vasudevan et al., 2015). Sequence and functional analysis of these alleles revealed several sites that are under positive selection and domains involved in recognition specificities (Ellis et al., 1999; Bhullar et al., 2009; Seeholzer et al., 2010). In case of rice blast resistance genes, it has also become evident that small sequence variations among individual *R* genes and between different *R* gene alleles often have a major functional impact on the resistance specificities. For example, eight amino acid changes differentiate resistance specificities of *Pi2* and *Piz-t* (Zhou et al., 2006), and a single amino acid change distinguish resistant and susceptible alleles of both *Pita* and *Pid2* (Bryan et al., 2000; Chen et al., 2006). Many recently cloned blast *R* genes such as *Pid3-A4*, *Pi54rh*, *Pi54of*, and *Pi35* are alleles/orthologs of known blast *R* genes (Das et al., 2012; Lv et al., 2013; Devanna et al., 2014; Fukuoka et al., 2014). These new alleles show varying patterns of resistance compared to the originally cloned *R* genes. Utilization of multiple alleles could increase the field durability of resistance by reducing the selection pressure on the pathogen (Fukuoka et al., 2014).

We have investigated the allelic diversity of *Pib* in rice accessions from 12 major rice-growing countries. *Pib* was the first cloned rice blast resistance gene. It was identified in the indica type Malaysian rice cultivar Engkatek and cloned from the near isogenic line Tohoku IL9 (Wang et al., 1999). *Pib* is an NBS-LRR type of *R* gene that confers broad spectrum resistance against a wide range of blast isolates present in South and Southeast Asian countries including Japan, China, Indonesia, and Korea (Wang et al., 2001; Yokoo, 2005; Roychowdhury et al., 2012). *Pib* is effective against *M. oryzae* race IE1k, which caused breakdown of the broad-spectrum blast *R* gene *Pita*, and race IB1 that is virulent against *Piz*, which also conferred stable blast resistance (Roychowdhury et al., 2012). We identified 25 new alleles of *Pib* in which most sequence variations are present in the NB-ARC and LRR domains. These new alleles represent a valuable genetic resource for rice blast resistance breeding programs and increase our understanding of the geographic dynamics of rice-*M. oryzae* interactions at the molecular level.

## Results

### Selection of Rice Genotypes for Mining *Pib* Allele Diversity

**FIGURE 1:**
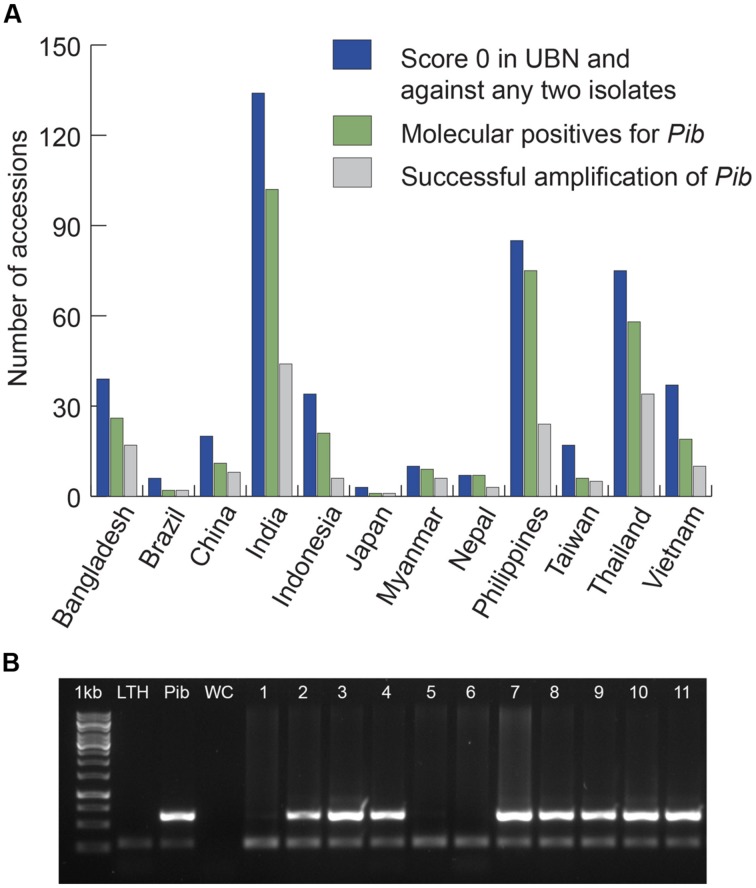
**Selection of candidates for *Pib* allele mining.** Accessions from 12 major rice growing countries that were chosen based on their phenotype (score 0) in UBN and against at least two of the five pure blast isolates are presented **(A)**. The accessions that are molecular positives for *Pib* based on Nsb marker screening were chosen as candidates, and the candidates from which *Pib* was successfully amplified are also presented. Sample picture of molecular screening of rice accessions for selection of *Pib* allele mining candidates is shown **(B)**. 1 kb, DNA marker; LTH, negative control; *Pib*, positive control (*Pib*-monogenic line); WC, water control; 1–11, test accessions. The accessions positive for *Pib* are 2 (IRGC-4471), 3 (IRGC-4552), 4 (IRGC-4553), 7 (IRGC-4574), 8 (IRGC-4619), 9 (IRGC-4633), 10 (IRGC-4634), and 11 (IRGC-4642). The upper band (629 bp) indicates the presence of *Pib* and the lower band (∼250 bp) shows the actin band, which is an internal control for the PCR.

We screened 4246 rice accessions originating from 13 major rice growing countries and identified 3176 blast-resistant accessions of which 2494 scored ‘highly resistant’ (score 0 on standard evaluation scale of 0–9 for leaf blast; Vasudevan et al., 2014). For *Pib* allele mining, we selected 467 rice accessions with a phenotypic score of 0 in a uniform nursery screening and against at least two of five tested *M. oryzae* isolates (**Figure 1**). These 467 accessions were from 12 of the 13 selected countries because none of the South Korean accessions met our scoring criteria. The selected accessions were screened for the presence of *Pib* (**Figure 1**) using the gene-specific Nsb molecular marker (Cho et al., 2007). A total of 337 rice accessions with the corresponding band of 629 bp for *Pib* were selected as candidates for *Pib* allele mining (**Figure 1**; **Supplementary Table S1**). The 337 accessions represent the three major rice varietal groups, indica (295), javanica (30), and japonica (12).

### Identification of New *Pib* Alleles

**FIGURE 2:**
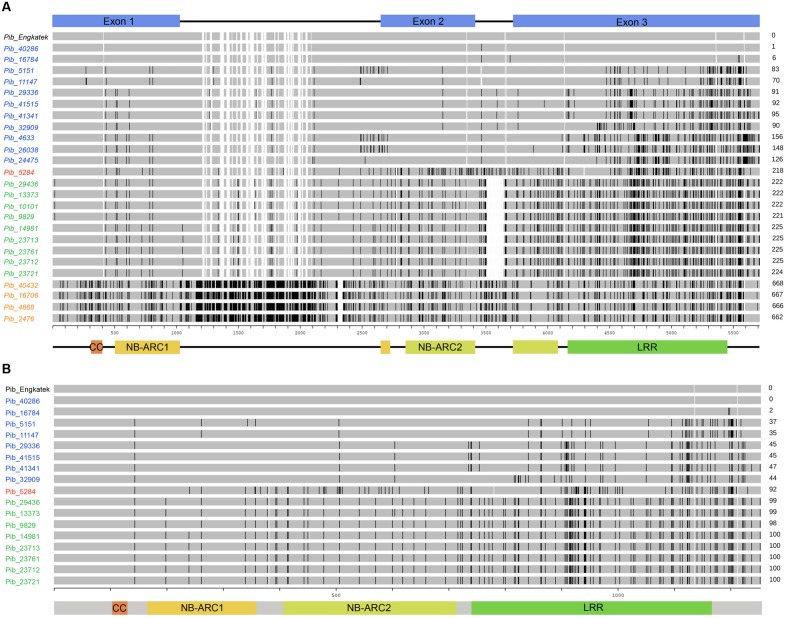
**Schematic representation of sequence alignments of newly identified *Pib* alleles.** The DNA sequence **(A)** and protein sequence **(B)** alignment of new *Pib* alleles is shown relative to the reference allele. The coding regions and the domains, i.e., CC, NB-ARC1, NB-ARC2, and LRR are illustrated at the bottom. The unit scale represents the nucleotide position **(A)** or AA position **(B)**. The black lines on the bars as well as the numbers on the right represent polymorphisms as compared to the reference. The alleles/proteins labeled in blue, green, orange, and red indicate category I, II, III, and IV, respectively. The empty gaps in the bars for some new alleles/proteins that are not present in the reference allele bar indicate deletions. The empty gaps in the reference allele bar together with some of the new alleles/proteins appear due to presence of insertions in other compared alleles.

Forward and reverse primers were designed to amplify the full-length *Pib* coding region of 5404 bp. The *Pib* gene was successfully amplified from 160 rice accessions (**Supplementary Table S2**) while the amplification was not successful in the remaining accessions, possibly due to sequence polymorphisms at the primer binding sites. Of these 160 accessions, 124 were indica, 24 javanica and 12 japonica accessions, and 47 of these accessions were landraces. Detailed sequence analysis using the Engkatek *Pib* sequence as reference allele (hereafter, referred to as *Pib_Engkatek*) revealed 25 new *Pib* alleles (**Table 1**). They were named after the IRGC accession from which the respective allele was first isolated in our study. **Figure 2A** summarizes the nucleotide polymorphisms in the 25 new *Pib* alleles as compared to *Pib_Engkatek*. The accessions from India, Thailand, and China comprise a relatively higher number of diverse *Pib* alleles with 10, 7, and 6 different *Pib* alleles identified in the accessions from these countries, respectively (**Table 2**). *Pib_23721* and *Pib_40286* are the most common *Pib* alleles that were identified in 35 and 30 accessions, respectively. Except for *Pib_41341*, *Pib_26038*, *Pib_24475*, *Pib_29436*, *Pib_23713*, *Pib_23712*, and *Pib_4868* that were found only in one rice accession, all other alleles were identified in at least two accessions (**Table 2**). None of the sequenced accessions had the *Pib_Engkatek* allele. The new *Pib* alleles share between 93 and 99% DNA sequence identity with *Pib_Engkatek* (**Table 2**).

**Table 1:** The IRGC accessions carrying the newly identified *Pib* alleles.

*Pib* alleles	IRGC accessions carrying the respective *Pib* allele
*Pib_40286*	40605; 18043; 18067; 20067; 20115; 20119; 25117; 812; 40286; 40287; 40293; 40296; 40300; 40303; 40304; 40333; 40334; 40405; 40413; 40418; 40423; 40457; 40459; 52922; 840; 15034; 24375; 24431; 24446; 24247
*Pib_16784*	16784; 32150
*Pib_5151*	49698; 5151; 49875
*Pib_11147*	11147; 32960
*Pib_29336*	29261; 29264; 29265; 29336; 29337; 29338; 29344; 29347; 32893; 41509; 41895; 42341
*Pib_41515*	41515; 41665; 42159
*Pib_41341*	41341
*Pib_32909*	32908; 32909
*Pib_4633*	4633; 5176
*Pib_26038*	26038
*Pib_24475*	24475
*Pib_5284*	21555; 40975; 42373; 42380; 42413; 5173; 5221; 5284; 5827
*Pib_29436*	29436
*Pib_13373*	25887; 13373
*Pib_10101*	5881; 10101
*Pib_9829*	5851; 9829; 26495; 26606; 3707; 4804; 5894; 9106; 10067; 43116; 46028; 46124; 16109
*Pib_14981*	8211; 8215; 8282; 14981
*Pib_23713*	23713
*Pib_23761*	22146; 5735; 17105; 23761; 27742; 48197
*Pib_23712*	23712
*Pib_23721*	14574; 21511; 21516; 21548; 21562; 22045; 22051; 22080; 22109; 22165; 22318; 22336; 22349; 22381; 22459; 22535; 22613; 7467; 7471; 8285; 15031; 15035; 23721; 23722; 23734; 23739; 23748; 23776; 27652; 27653; 27658; 27661; 27702; 27719; 27732
*Pib_40432*	40430; 40432
*Pib_16706*	38994; 973; 49496; 21566; 21639; 23780; 5612; 9786; 7104; 16706; 16768; 24129; 32058; 32079; 32080
*Pib_4868*	4868
*Pib_2476*	735; 4574; 2476; 6786; 33446; 33762; 5493; 5528; 5536


*New alleles identified in this study have been named after the IRGC accession from which the respective allele was first isolated in this study. The accessions chosen to represent the respective allele of *Pib* are indicated in blue.*

**Table 2:** Detailed information regarding the newly identified *Pib* alleles and the accessions carrying the respective alleles.

*Pib* alleles	% identity to reference	Unique SNPs/InDels	Origin of accessions carrying the allele	Number of accessions carrying the allele		
				
				Indica	Japonica	Javanica
*Pib_24475*	98	^∗^	Thailand	1	-	-
***Pib_41515***	98	^∗^	India	3	-	-
***Pib_41341***	98		India	1	-	-
***Pib_32909***	98	^∗^	Bangladesh	2	-	-
***Pib_11147***	99	^∗^	Myanmar	2	-	-
***Pib_16784***	99	^∗^	Vietnam	1	-	1
*Pib_26038*	97	^∗^	Brazil	-	1	-
***Pib_29336***	98		Bangladesh, India	12	-	-
*Pib_4633*	97	^∗^	China, Philippines	2	-	-
***Pib_5151***	98	^∗^	China, India	3	-	-
***Pib_40286***	99		China, Thailand, Vietnam, Indonesia, Philippines	29	1	-
***Pib_14981***	98	^∗^	Thailand	-	3	1
***Pib_23713***	98		Thailand	-	-	1
***Pib_23712***	98	^∗^	Thailand	-	-	1
*Pib_10101*	98	^∗^	India	2	-	-
***Pib_29436***	98		Bangladesh	1	-	-
***Pib_13373***	98		Bangladesh, Nepal	2	-	-
***Pib_23721***	98		India, Thailand	12	5	18
***Pib_9829***	98		Bangladesh, India, Nepal	13	-	-
***Pib_23761***	98	^∗^	India, Myanmar, Thailand	4	-	2
*Pib_4868*	93		China	1	-	-
*Pib_40432*	93		Philippines	2	-	-
*Pib_2476*	93	^∗^	Myanmar, China, Taiwan, Japan	9	-	-
*Pib_16706*	93		Brazil, China, India, Nepal, Taiwan, Vietnam	13	2	-
***Pib_5284***	96		India, Philippines	9	-	-


*Asterisk indicates alleles with unique SNPs and/or InDels. Alleles indicated in bold possess a complete ORF as that of reference allele.*

### Sequence and Structure Diversity of New *Pib* Alleles

The *Pib* alleles were grouped into four allele categories based on the characteristics of their intron regions and nucleotide polymorphisms relative to the *Pib_Engkatek* reference allele (**Figure 2A**). Category I alleles (*Pib_40286*, *Pib_16784*, *Pib_5151*, *Pib_11147*, *Pib_29336*, *Pib_41515*, *Pib_41341*, *Pib_32909*, *Pib_4633*, *Pib_26038*, and *Pib_24475*) have no long sequence insertion/deletions (InDels) in their introns. Category II alleles (*Pib_29436*, *Pib_13373*, *Pib_10101*, *Pib_9829*, *Pib_14981*, *Pib_23713*, *Pib_23761*, *Pib_23712*, and *Pib_23721*) have a 136 nucleotide deletion in intron 2. Category III alleles (*Pib_40432*, *Pib_16706*, *Pib_4868*, and *Pib_2476*) have several insertions of varying lengths ranging from one to 28 nucleotides and a deletion of 42 nucleotides in intron 1. Category IV has only one allele, *Pib_5284*, without large InDels but unique nucleotide differences relative to *Pib_Engkatek*. *Pib_5284* introns and exons partially share the sequence polymorphisms found in category I, II and III alleles (**Figure 2A**), suggesting that *Pib_5284* is an intermediate allele of the other *Pib* allele categories.

Seventeen of the *Pib* alleles have complete open reading frames (ORFs) similar to that of *Pib_Engkatek* (**Figure 2B**). The remaining eight alleles have ORFs of variable lengths due to premature stop codon(s) resulting from point mutations and/or InDels. The ORFs of all category III alleles are disrupted by insertions that cause frame shifts or introduce alternative splicing sites. Category I alleles *Pib_40286* and *Pib_16784* have only minor nucleotide polymorphisms compared to *Pib_Engkatek*. *Pib_40286* differs from *Pib_Engkatek* by a single nucleotide change at position 3166 in intron 2. This nucleotide change is shared among all *Pib* alleles except *Pib_5151* and *Pib_11147*, which both have an eight bp deletion (**Figure 2A**). Except *Pib_40286* and *Pib_16784*, all the other alleles have several nucleotide polymorphisms that are partially or completely shared within and/or between the four *Pib* allele categories (**Figure 2A**). Such partially or completely shared nucleotide polymorphisms indicate frequent sequence exchange between the alleles, possibly through gene conversion events. Thirteen alleles have one or more unique single nucleotide polymorphisms (SNPs) and/or InDels that are absent in any other *Pib* allele (**Table 2**). *Pib_5284* has the most unique SNPs (**Figure 2A**). Nine of these 13 alleles were found in accessions of a particular country of origin (**Table 2**). The remaining four alleles (*Pib_5151*, *Pib_4633*, *Pib_23761*, and *Pib_2476*) were found in accessions that originate from different but geographically close countries (e.g., *Pib_5151*: China and India; **Table 2**). This suggests that such unique SNPs might be specific for resistance to prevailing *M. oryzae* races in certain geographical locations.

### *Pib* Alleles Have Domain-Specific Sequence Polymorphisms Indicating Balanced and Positive Selection

The *Pib* N-terminal CC domain sequences of all category I, II, and IV alleles are highly conserved and have no SNPs or InDels, but category III alleles have nine SNPs (**Figure 2A**). *Pib* encodes two NB-ARC domains that are interrupted by intron 1 (1015–2354) and intron 2 (3111–3418) located 87 nucleotides 3′ of a kinase 3a motif in both NB-ARC domains. The NB-ARC domains of R proteins are involved in plant defense signaling. The NB-ARC-1 domains of category I (10 SNPs), category II (nine SNPs), and category IV (nine SNPs) alleles have fewer nucleotide changes compared to the NB-ARC-1 domains of category III alleles, which have 30 SNPs in this domain. The NB-ARC-2 domains of category II, III and IV alleles have between 35 and 52 SNPs, a three-nucleotide deletion in *Pib_14981*, and a three-nucleotide insertion in all category III alleles. In contrast, category I alleles have only five SNPs in this domain (**Figure 2A**).

**FIGURE 3:**
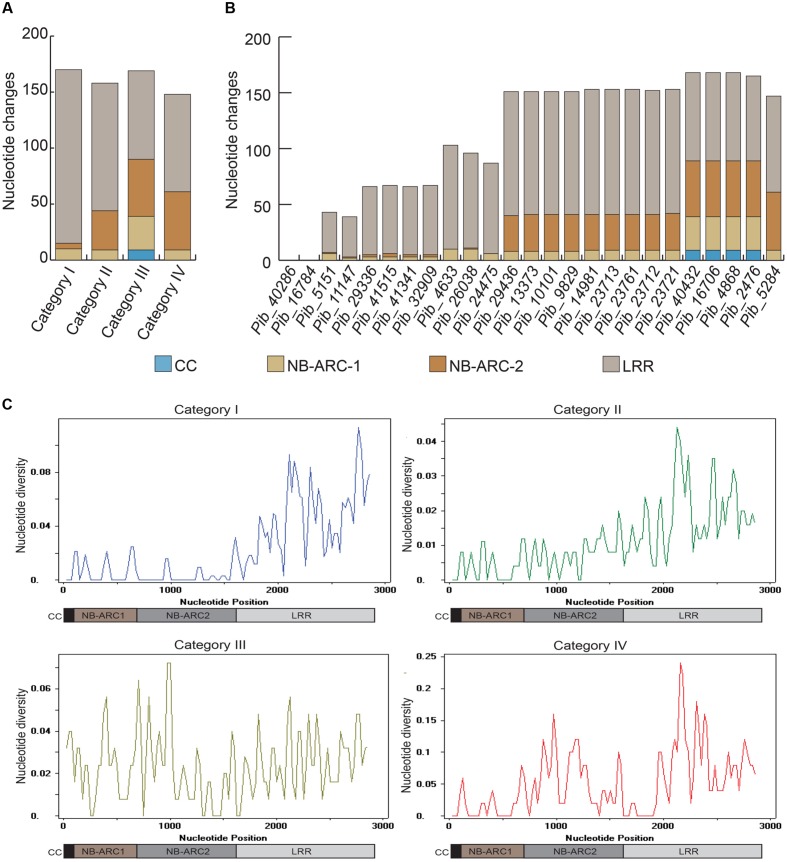
**Comparison of nucleotide diversity among different domains of *Pib* alleles.** Graphical representation of overall domain-based nucleotide changes in each of the four *Pib* categories are shown **(A)**, together with domain based nucleotide changes in each of the newly identified *Pib* alleles **(B)**. The sliding-window analysis of nucleotide diversity (π) in different domains of *Pib* alleles is shown **(C)**. The domains are illustrated below the unit scale that represents nucleotide positions.

The LRR domains of *R* genes are known to be involved in protein-protein interactions and in determining the resistance specificities. The *Pib* LRR domain sequences have the most nucleotide changes in the majority of the alleles. Based on the number of LRR nucleotide changes calculated as a percentage of total coding region nucleotide changes, category I alleles have the highest LRR diversity (67.1%), followed by category II (58.2%), category IV (46.9%), and category III (35.9%) alleles. Most of these nucleotide changes cause amino acid changes in the deduced protein sequence (discussed below). In case of category I and category II alleles, the highest nucleotide diversity is confined to the LRR, whereas, for category III and category IV alleles nucleotide diversity is distributed similarly between NB-ARC and LRR domain sequences (**Figure 3A**). Although the number of LRR polymorphic sites is very high in category I alleles, they are only partially shared among the alleles. In contrast, category II LRR polymorphisms are completely shared among the alleles (**Figures 2A** and **3B**). Together, the polymorphism in the NB-ARC and LRR domains of *Pib* alleles indicate various levels of selection pressures promoting the evolution of new resistance specificities in these alleles.

Sequence variations were also found in the two different *Pib* zinc finger motifs TTF and U1 (Gupta et al., 2012). The TTF motif sequence in exon 1 (positions 178–426) spans the CC domain while the U1 motif (positions 2976–3080) lies within the NB-ARC2 domain sequence in exon 2. TTF sequences of category I, II, and IV alleles are conserved with only two synonymous mutations in category I alleles *Pib_5151* and *Pib_11147* and one synonymous mutation in category IV allele *Pib_5284*. Category III alleles have 21 SNPs and a three-nucleotide insertion in their TTF motif. The U1 motif is conserved in category I alleles, whereas, 3, 4, and 10 SNPs were observed in category II, III, and IV alleles, respectively. In addition, a triple nucleotide insertion was found in the U1 motif of category III alleles. The Zinc finger domains determine the regulatory function of NBS-LRR proteins (Gupta et al., 2012), and our results further indicate their significant role in determining *Pib* function.

Domain-specific sequence analysis using DnaSP revealed selection patterns and divergence in the four allele categories. The number of mutations (η) was higher than the number of polymorphic/segregating sites (S) only in category I alleles (**Table 3**). Moreover, all segregating sites with more than one mutation are located within the LRR domain of category I alleles (S/LRR = 146, η/LRR = 155; **Table 3**). S and η values of category II and III alleles were the same, and category IV has only one allele. Sliding window analysis of nucleotide diversity (π) in each allele category showed a high rate of diversity in the LRR domain for category I, NB-ARC2 and LRR domains for category II and IV, and in all domains for category III alleles (**Figure 3C**). Tajima’s D value suggests that category I alleles are under balancing selection, whereas, category II and III are under positive selection (**Table 3**).

**Table 3:** Nucleotide diversity of the 25 new *Pib* alleles.

	*Pib* alleles	Number of segregating sites (S)	Number of mutations (η)	Nucleotide diversity (π)	Tajima’s D test
Full length	Overall	916	957	0.05495	0.54639
	Category I	235	252	0.01761	0.64727
	Category II	220	220	0.00899	-1.95664
	Category III	602	603	0.04526	-1.23986
	Category IV	211	211	0.03915	^∗^
Exons only	Overall	439	471	0.04046	0.905
	Category I	214	231	0.0232	0.65257
	Category II	196	196	0.01078	-2.07968
	Category III	220	220	0.02375	-1.18486
	Category IV	183	183	0.0488	^∗^
CC	Overall	9	9	0.02708	0.10683
	Category I	-	-	-	-
	Category II	-	-	-	-
	Category III	9	9	0.04	-1.18441
	Category IV	-	-	-	^∗^
NB-ARC1	Overall	40	41	0.02075	0.4683
	Category I	10	10	0.00807	1.72143
	Category II	9	9	0.0037	-1.41191
	Category III	30	30	0.02062	-1.24614
	Category IV	9	9	0.01546	^∗^
NB-ARC2	Overall	89	93	0.03002	0.51095
	Category I	5	5	0.00174	-0.11051
	Category II	35	35	0.00763	-2.098
	Category III	51	51	0.02237	-1.19639
	Category IV	52	52	0.05646	^∗^
LRR	Overall	204	223	0.05839	1.09832
	Category I	146	155	0.04525	0.60669
	Category II	114	114	0.0182	-2.09205
	Category III	79	79	0.02514	-1.14421
	Category IV	86	86	0.06729	^∗^


*CC, coiled coil; NB-ARC, nucleotide-binding adaptor shared by APAF-1, R proteins, and CED-4; and LRR, leucine rich repeats. ^∗^Tajima’s D test not performed because of only one allele in this category.*

### *Pib* Proteins Have Conserved Structural Domains and Post-translational Modification Sites

Protein predictions and analysis were performed only for the 17 *Pib* alleles with complete ORFs similar to the *Pib_Engkatek* reference allele (**Table 2**). Some of the alleles differ only by intron mutations and therefore have identical protein sequences (Pib_40286/Pib_Engkatek, Pib_29336/Pib_41515, and Pib_23713/Pib_23721; **Figure 2B**). In some alleles, the 3-nucleotide InDels (described above) result in addition or deletion of an amino acid (AA) without disrupting the ORF. The category I proteins Pib_29336, Pib_41515, Pib_41341, and Pib_32909 have an additional isoleucine and arginine at AA positions 1136 and 1211, respectively. Category II Pib proteins have deletions of lysine (in all category II proteins) and aspartate (only in Pib_14981) at AA positions 382 and 453, respectively. Similarly, the category IV Pib_5284 protein has deletions of lysine and histidine at AA positions 382 and 780, respectively (**Figure 2B**).

**FIGURE 4:**
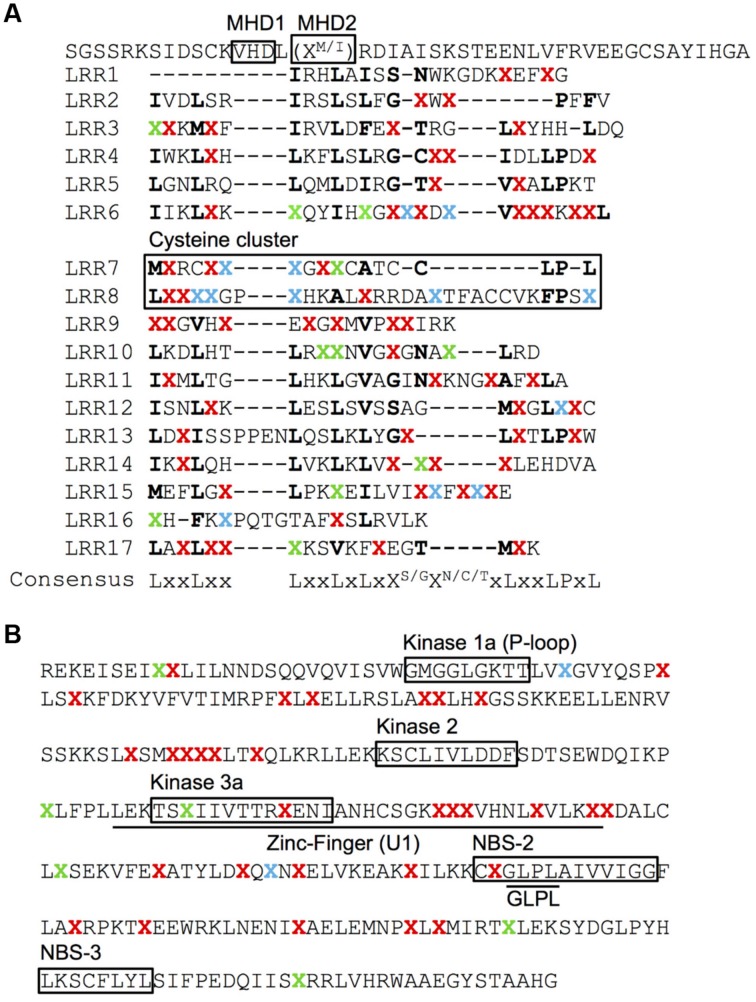
**Surface accessibility prediction of the Pib proteins.** Surface accessibility of AA of LRR **(A)** and NB-ARC2 **(B)** domains of *Pib* proteins was predicted using NetSurfP server. The LRR repeats **(A)** are indicated as initially described for *Pib* (Wang et al., 1999). ‘X’ represents polymorphic AA identified at least in one of the analyzed *Pib* proteins. The conserved AA are represented by their respective single letter code. The polymorphic AA predicted as solvent exposed are highlighted in red and the ones in green are predicted as buried. The polymorphic AA that are predicted as either exposed in some proteins or buried in rest of the analyzed proteins are highlighted in blue. The cysteine cluster of LRR and the functional motifs are indicated on the figure **(A,B)**.

No AA changes were found in the CC domain of the *Pib* proteins. The CC domain of several R proteins contains a functional EDVID motif (Rairdan et al., 2008; Wang et al., 2015). The *Pib* has two possible EDVID motif variants that are EDSLQ (80–84) and EDVSQ (120–124), the former located before the CC domain and the later within the CC domain. Both the EDVID motif variants are conserved among all the analyzed *Pib* proteins. The NB-ARC1 domain has five polymorphic AA residues in category I, II and IV *Pib* proteins. In the NB-ARC2 domain, category I *Pib* proteins have three AA changes, but category II and IV proteins have 20 and 31 AA changes, respectively (**Figure 2B**). Despite these AA changes, the Kinase 1a (P-loop), Kinase 2 and Kinase 3a motifs within the NB-ARC1 and NB-ARC2 domains are highly conserved in most *Pib* proteins. The GLPL (619–622) motif and the MHD motifs are also conserved among the *Pib* proteins (**Figure 4**). There are two MHD motifs in *Pib* (750–756) separated by a single amino acid. The MHD1 (VHD in case of *Pib*) is highly conserved in all *Pib* proteins (**Figure 4A**), while the MHD2 is identified as MRD in most of the category I (except Pib_29336, Pib_41515, and Pib_41341) and category IV *Pib* proteins, and as IRD in all of the category II *Pib* proteins. The higher number of SNPs in the LRR domain result in 56, 65, and 46 AA changes in category I, II and IV *Pib* proteins, respectively (**Figure 2B**), which account for 65, 58, and 44% of the total AA changes in these proteins, respectively. Among the category I proteins, the highest number of AA changes were found in Pib_29336, Pib_41515, Pib_41341, and Pib_32909. The LRR of Pib_5151, Pib_11147, and Pib_32909 has several unique AA changes that are not shared with other category I alleles. However, in category II, most AA changes are shared between the *Pib* proteins (**Figure 2B**). According to NetSurfP (Petersen et al., 2009) most of the polymorphic AA changes in the NB-ARC2 (79.5%) and LRR (71.4%) domains are solvent exposed residues (**Figure 4**), suggesting that these AA changes might be critical for interaction with *M. oryzae* effector proteins and/or other cellular proteins. However it is to be noted that, the solvent exposed residue predictions are not supported by detailed structural analysis.

According to ‘PredictProtein,’ on average 25 protein-binding sites are predicted in all *Pib* proteins, with minor changes in their positions among the different proteins (**Supplementary Figure S1**). Apart from the disordered regions found in the N- and C-terminal regions of most *Pib* proteins, two conserved disordered regions are detected between the Kinase 1a and Kinase 2 domains of both the NB-ARC-1 and NB-ARC-2, respectively (**Supplementary Figure S1**). In addition, all category I *Pib* proteins have a conserved disordered region between their NB-ARC-1 and NB-ARC-2 domains that is absent in category II and IV *Pib* proteins as the result of an AA replacement (Ala to Val) at position 378 and loss of a lysine at position 381.

Analysis of *Pib* protein sequences using ScanProsite revealed several conserved patterns of post-translational modification sites (PTM), including potential tyrosine kinase (4), casein kinase II (26), cAMP- and cGMP-dependent protein kinase (2) and protein kinase C phosphorylation sites (26), myristoylation sites (11), glycosylation sites (4), and amidation site (1) in Pib_Engkatek (**Table 4**). The tyrosine kinase phosphorylation sites are the only PTM sites that are highly conserved among all the *Pib* proteins, indicating that they are functionally relevant. The amidation site was found only in Pib_40286, Pib_16784, Pib_5151, Pib_11147, and Pib_5284. The other PTM sites were found in all *Pib* proteins, however, their numbers vary between different proteins when compared to Pib_Engkatek (**Table 4**). These predicted PTM sites and their differences among the *Pib* proteins might influence the functional structure of the protein.

**Table 4:** Post-translational modification sites in Pib proteins predicted using ScanProsite server.

Pib proteins	TKP	CK2 phosphorylation	cAMP/cGMP dependent PKP	PKC phosphorylation	*N*-myristoylation	*N*-glycosylation	Amidation
Pib_Engkatek	4	26	2	26	11	4	1
Pib_40286	4	26	2	26	11	4	1
Pib_16784	4	26	2	26	11	4	1
Pib_5151	4	26	2	25	12	3	1
Pib_11147	4	26	2	26	12	3	1
Pib_29336	4	24	2	24	11	3	-
Pib_41515	4	24	2	24	11	3	-
Pib_41341	4	24	2	24	11	3	-
Pib_32909	4	23	2	26	11	3	-
Pib_5284	4	27	3	24	12	4	1
Pib_29436	4	23	2	27	9	4	-
Pib_13373	4	23	2	27	9	4	-
Pib_9829	4	23	2	27	9	4	-
Pib_14981	4	23	2	26	9	4	-
Pib_23713	4	23	2	27	9	4	-
Pib_23761	4	23	2	27	9	4	-
Pib_23712	4	23	2	27	9	4	-
Pib_23721	4	23	2	27	9	4	-


*TKP, tyrosine kinase phosphorylation; CK2, casein kinase II; PKP, protein kinase phosphorylation; PKC, protein kinase C.*

**FIGURE 5:**
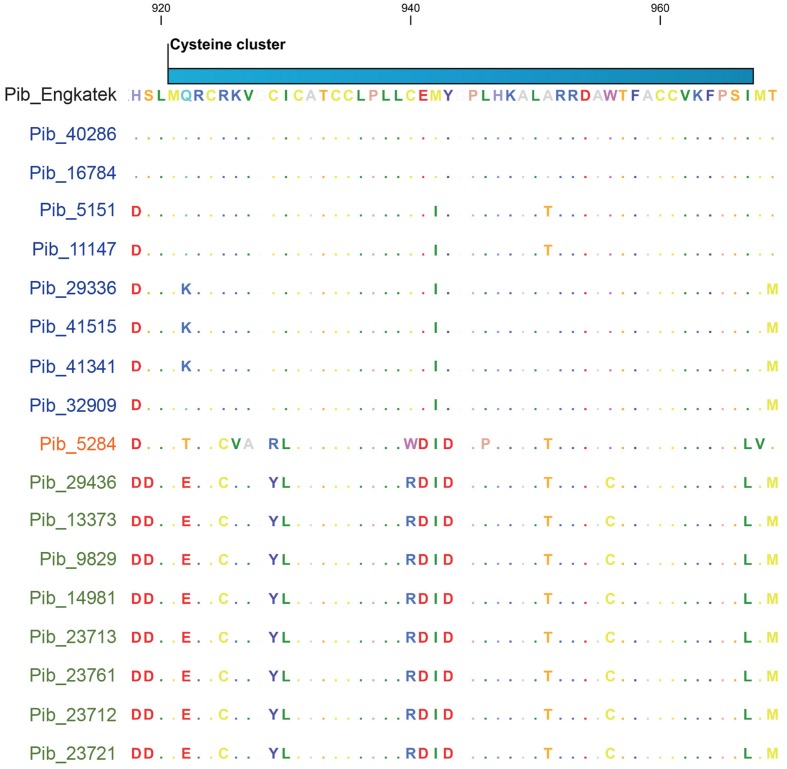
**Selection window of cysteine-cluster region from the Pib protein sequence alignment.** Proteins labeled in blue, orange, and green represents *Pib* category I, IV, and II, respectively. Scale on top indicates the amino acid position.

The cysteine cluster in the center of the *Pib* LRR (AA positions 921–967) containing eight cysteine residues (Wang et al., 1999) is highly conserved in the category I *Pib* proteins. Six of the eight cysteines are also conserved in the category II *Pib* proteins, and the two additional cysteines at positions 925 and 956 may compensate the loss of the cysteines at AA positions 929 and 940 (**Figure 5**). The cysteine cluster of the category IV Pib_5284 has also lost two conserved cysteines similar to category II *Pib* proteins but compensated these with the addition of two cysteines at AA positions 925 and 1228. This strong conservation of seven or eight cysteines in the center of the LRR suggests that the cysteine cluster in the *Pib* proteins has a structural and/or functional role.

### Distribution and Genetic Relatedness of *Pib* Alleles from Diverse Geographical Origins

**FIGURE 6:**
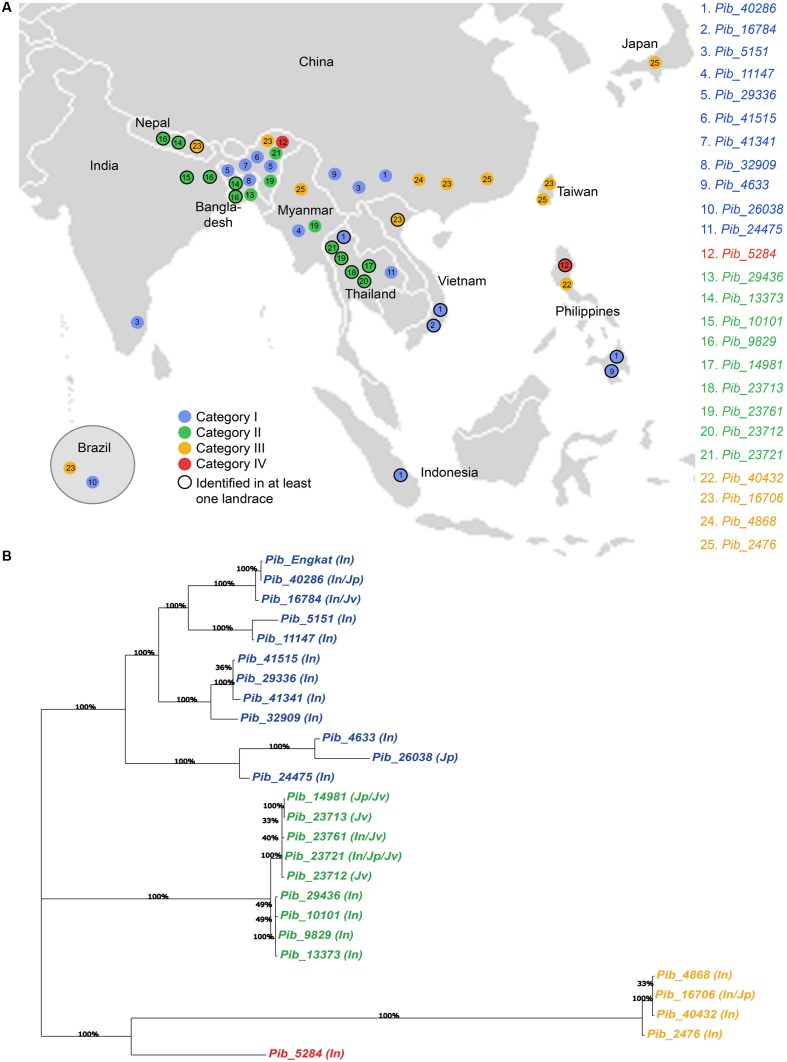
**Geographical distribution of newly identified *Pib* alleles and their genetic relatedness.** Map view of geographical distribution of newly identified *Pib* alleles **(A)**. The accessions for which the information on local collection site is available in the International Rice Germplasm Collection Information System, were placed on the map accordingly. Phylogenetic analysis was performed with DNA sequences of *Pib* alleles together with *Pib_Engkatek* to analyze their genetic relatedness **(B)**. The varietal group for accessions carrying each allele is mentioned in brackets, i.e., In, indica; Jp, japonica and Jv, javanica. Bootstrap values (100 replications) are mentioned at the branch nodes. The alleles labeled in blue, green, orange, and red represent category I, II, III, and IV, respectively.

Sixteen of the 25 *Pib* alleles were observed only in the indica accessions, whereas, only one and two alleles were found exclusively in the japonica and javanica accessions (**Table 2**). To some extent this may reflect the sampling number of accessions in the rice subspecies, but could also indicate a higher dynamics in the co-evolution of *M. oryzae* with the indica subspecies. Among the 25 *Pib* alleles, 13 alleles were identified in at least one landrace. Different landraces that have the identical *Pib* allele mostly originated from the same country, except for those having *Pib_40286*, *Pib_13373*, *Pib_9829*, and *Pib_16706* alleles. The landraces having these four alleles originated from different countries, but most of them are in geographic proximity. For example, *Pib_13373* is present in landraces from Bangladesh and Nepal, and *Pib_9829* in landraces from India and Bangladesh (**Supplementary Table S2**). Except Indonesia and Japan, all other countries have accessions with more than one new *Pib* allele. Fourteen *Pib* alleles are country specific, with Thailand (4), India (3), and Bangladesh (2) having more than one such origin-specific alleles. None of the accessions from Indonesia, Japan, Nepal and Taiwan had such origin-specific allele (**Table 2**). The geographical origin of the new *Pib* alleles (**Figure 6A**) showed that category I alleles are widely distributed in Southeastern Asia. Of the category II alleles, three are present in Northeast India, Nepal and Bangladesh, while five are predominantly found in Thailand. The category III alleles were distributed in the Eastern Asia while the category IV allele is present only in accessions from India and the Philippines (**Figure 6A**).

Phylogenetic analysis of the sequence polymorphisms of the 25 *Pib* alleles and *Pib_Engkatek* revealed that category I, II III, and IV alleles form distinct clusters and that category III alleles are phylogenetically distant (**Figure 6B**). Most of category I and III alleles are found in indica sub-species, whereas, in category II the alleles of one sub-cluster are found exclusively in indica sub-species while the alleles in the other sub-cluster are found in javanica and japonica sub-species (**Figure 6B**). When comparing the LRR domain only, category II alleles are phylogenetically distinct from all other alleles. The LRR of category III alleles is closely related to category I alleles than to category II, while the category IV allele *Pib-5284* clusters among the category I alleles (**Supplementary Figure S2**). In case of NB-ARC-1 domain only, the category III alleles cluster together with category I alleles, while the category IV allele clusters within the category II cluster forming a separate branch. While, in case of the NB-ARC-2 domains only, the overall tree-structure and clustering remains similar to that for the entire coding sequence analysis, except for a few changes in the sub-clustering of category I alleles (**Supplementary Figure S2**). These results suggest frequent sequence shuffling and gene conversion events at the *Pib* locus that create natural allele chimeras by inter-allele recombination, possibly leading to the expansion of a functional allele series and/or gene diversification.

## Discussion

The extensive use of high-yielding varieties has greatly reduced the genetic diversity in breeding germplasm of major food crops, which limits future crop improvements (Tanksley and McCouch, 1997; Warschefsky et al., 2014). The analysis of nucleotide variation in *Oryza sativa* and its wild relatives found that only 20 and 10% of the diversity in wild rice species were retained in the cultivated indica and japonica rice sub-species, respectively (Zhu et al., 2007). Large-scale cultivation of genetically uniform varieties makes them more vulnerable to disease epidemics because of the high selection pressure on the pathogen populations. Because of changing and evolving pathogen populations, crop plant diseases are continuous problems that are influenced by various factors including selection pressure, environmental conditions and the changing climate. Consequently, crop protection requires continuous improvements to keep pace with pathogen evolution and disease severity. This implies the need for new resistance genes and alleles beyond those available in the breeding gene pool, such as genes and alleles from landraces and wild relatives. Much of the genetic diversity of crop plants is being maintained in the seed banks that have large collections of germplasm of diverse origins, but most accessions that are not yet fully annotated and their genotypic diversity is not well-understood (McCouch et al., 2012). We explored the allele diversity of *Pib* in 467 rice accessions originated from 12 major rice-growing countries and identified 25 new *Pib* alleles that represent one of the largest allelic series known among blast *R* genes.

Among the 25 *Pib* alleles, 14 alleles are country specific, suggesting the existence of distinct *M. oryzae* populations in different geographical regions. Accordingly, currently unknown selective pressures are causing the co-evolution of *Pib* alleles with sequence polymorphisms specific to a geographical region. In addition to SNPs that are partly or fully shared within or between the four *Pib* allele categories, several unique SNPs/InDels were identified in 13 of the *Pib* alleles. The shared SNPs likely resulted from gene conversion events and the unique polymorphisms could be the result of selection pressure specific to the *M. oryzae* populations in a particular geographical location. Studies on various *M. oryzae* populations collected from different parts of China and allelic variation among *AvrPib* alleles indicated that *Pib* shaped the genetic architecture of *M. oryzae* populations (Zhang et al., 2015). It is also reported that some rare *AvrPib* types were restricted to *M. oryzae* populations in Southern China as compared to those in Northeastern China, particularly indicating host selection pressure owing to *Pib* donor cultivars in these climatically distinct regions of China (Zhang et al., 2015). However, the effects of the unique SNPs/InDels observed in the *Pib* alleles need to be experimentally verified through functional validation and comparison of resistance specificities of alleles with shared SNPs and that of alleles with additional unique polymorphism(s). These different *Pib* alleles could be subjected to interaction studies against recently reported *AvrPib* and its allelic variants (Zhang et al., 2015), as such analysis would help in further strengthening our understanding of the molecular interactions between *Pib* and AvrPib as well as their co-evolution.

Nucleotide diversity, positive selection sites and selection pressure on *R* gene alleles can be assigned either to a single or more domains. For example, specificity differences between alleles of the flax rust resistance gene *L* can be determined by both the LRR and TIR regions, whereas, for the barley powdery mildew resistance gene *Mla*, most of the positive selection sites are found within the LRR of functional alleles (Ellis et al., 1999; Seeholzer et al., 2010). Similarly, for *Pib* we identified alleles in which diversity and selection pressure appears to be restricted to either LRR (category I) or NB-ARC2 and LRR (category II and IV) and in which all domains are under selection pressure (category III). Together, our results suggest that diversified selection events have occurred at the *Pib* locus. Moreover, category I alleles have higher diversity (unique SNPs), variable segregation sites between alleles, η > S and a positive Tajima’s D value. This indicates that category I alleles are under either balancing selection or, more likely, divergence selection, eventually resulting in further divergence, which is supported by the phylogenetic sub-clustering of alleles (**Figure 6B**).

The LRR domain determines the resistance specificity of several R proteins and physically interacts with cognate effector proteins. Based on the functional analysis of R proteins such as *Arabidopsis* RPP1 (Krasileva et al., 2010), wheat Pm3 (Bhullar et al., 2009, 2010), flax L (Ellis et al., 1999; Ravensdale et al., 2012) and barley Mla (Seeholzer et al., 2010), the LRR domain is necessary and in some cases sufficient to maintain the same recognition specificity as that of the full-length R protein (Krasileva et al., 2010). It was shown that replacing six amino acids in the LRR domain of flax *P2* can confer *P1* specificity (Dodds et al., 2001). The recognition of AvrL567 by L6 is LRR controlled, although, the TIR domain can affect the specificity of pathogen recognition (McHale et al., 2006; Ellis et al., 2007). We found a high rate of nucleotide polymorphisms in the LRR region of *Pib* alleles. If only the exon regions of the 25 alleles are considered, then 47% of the total nucleotide polymorphisms are within the LRR domain, resulting in 55% of the total amino acid changes. This suggests that the *Pib* LRR domain has a significant role in establishing recognition specificities for *M. oryzae* effector proteins. In addition, the cysteine cluster in the center of the *Pib* LRR (Wang et al., 1999) is strongly maintained despite the loss and gain of two cysteine residues as the result of AA changes in the category II and IV proteins. Although the function of the cysteine cluster is still unknown, it likely has a structural and/or functional role in the *Pib* protein.

The CC domain is also proposed to have an important role in R protein functioning (Bai et al., 2012; Hao et al., 2013; Zhai et al., 2014), and the EDVID motif is reported to be important for intramolecular interaction and function of CC domain (Rairdan et al., 2008; Wang et al., 2015). The CC domain and the two EDVID motif variants in/around CC domain are conserved among the *Pib* proteins, however, it is not known which of the two EDVID motif variants is critical for its function. The NB-ARC domain functions as molecular switch that stabilizes the functional structure of R proteins (McHale et al., 2006; Tameling et al., 2006; Williams et al., 2011). The disordered regions within and between the two *Pib* NB-ARC domains could also facilitate intramolecular interactions that stabilize the proteins. In Pid3, functional AA polymorphisms between resistant and susceptible orthologs are located in their Kinase 1a motif of NBS domains (Xu et al., 2014). In Pib, all Kinase motifs in category I proteins are highly conserved. Similarly, despite several AA changes in the NB-ARC domains of category II and IV *Pib* proteins, the sequences in the Kinase 1a, Kinase 2, and Kinase 3a motifs within these two domains are highly conserved, including sequences critical for ATP hydrolysis (McHale et al., 2006; Tameling et al., 2006; Chattopadhyaya and Pal, 2008). It is therefore unlikely that the minor AA changes we found in some of the category II and IV Kinase motifs would result in *Pib* loss-of-function proteins. The GLPL and MHD motifs of the NB-ARC domains have also been shown to affect the function of R proteins (van Ooijen et al., 2008; Williams et al., 2011; Wang et al., 2015). The GLPL motif located in the NB-ARC2 domain of *Pib* is highly conserved among all *Pib* proteins. Among the MHD motifs, the MHD1 variant (VHD) is highly conserved but the MHD2 is identified in two different forms, MRD or MID. The histidine and aspartate are the most conserved residues of MHD1 in most of the plant R proteins (van Ooijen et al., 2008) and aspartate is the most conserved residue in case of MHD2 (Wang et al., 2015). The observation of conserved residues of MHD1 and MHD2 motifs of *Pib* is consistent with these reports.

Most of the positive selection sites in Mla proteins are solvent-exposed AA clustered in the LRR domain (Seeholzer et al., 2010). Similarly, among the category I, II, and IV *Pib* proteins, 79.5 and 71.4% of the polymorphic AA in NB-ARC2 and LRR domains, respectively, are predicted to be solvent-exposed. This suggests that surface-exposed polymorphic AA in *Pib* proteins might have functional roles such as interactions with one or more effector proteins or metabolites that establish resistance specificities. However, these solvent-exposed residue predictions are only sequence-based and not supported by in depth structure analysis. To validate such functional roles requires complementation assays and/or screening against blast isolates from various geographical locations. Once validated, the distinct polymorphisms among the *Pib* alleles can be combined to generate novel resistance specificities. For example, the resistance specificities of wheat *Pm3d* and *Pm3e* alleles were successfully fused into a single chimeric allele by intragenic allele pyramiding (Brunner et al., 2010). Similarly, a recombinant flax rust resistance allele *L2-L10* has a novel recognition specificity that is distinct from the *L2* and *L10* parental alleles (Ellis et al., 1999). The detailed understanding of *Pib* alleles and polymorphisms that contribute to differences in resistance specificity to *M. oryzae* isolates will enable us to combine specificities through inter-allelic pyramiding or using CRISPR/Cas-mediated editing of polymorphic sites.

## Materials and Methods

### Plant Material and Molecular Screening for *Pib*

The rice germplasm material was obtained from the International Rice GenBank (IRG) of the International Rice Research Institute (IRRI), Philippines. A set of 467 rice accessions with a phenotypic score of 0 in a uniform nursery screening and against at least two of five tested *M. oryzae* isolates (Vasudevan et al., 2014) was chosen for *Pib* allele mining. The isolates were selected based on their virulence pattern on near isogenic lines for major rice blast resistance genes (Vasudevan et al., 2014) and it is not known if these isolates have a functional *AvrPib.* Molecular screening for identifying the accessions carrying the *Pib* gene was carried out using the gene specific marker Nsb (Cho et al., 2007; Forward: 5′-ATCAACTCTGCCACAAAATCC; Reverse: 5′-CCCATATCACCACTTGTTCCCC). Genomic DNA extracted from a *Pib* monogenic line was used as a positive control and that from susceptible rice cultivar LTH was used as the negative control, in addition to a water control. Additionally, the primers for amplification of the rice actin gene were used in PCR reaction mixtures as an internal PCR control (Forward: 5′-TTATGGTTGGGATGGGACA; Reverse: 5′-AGCACGGCTTGAATAGCG).

### Isolation and Cloning of *Pib* Alleles

The forward primer 5′-CCACAAAATCCATTCAAAAATAGA ACAGAGCA and reverse primer 5′-GGAGCACGGCAAAGT AACTCCAAAGGAG were used to amplify the full-length coding region of *Pib*. PCR was performed with an initial denaturation at 95°C for 5 min; followed by additional denaturation at 98°C for 20 s, annealing at 63°C for 20 s, extension at 72°C for 3 min (these three steps repeated for 35 cycles); followed by final extension at 72°C for 6 min, using KAPA HiFi HotStart DNA polymerase (high fidelity proof-reading enzyme). The samples yielding low amplified product were repeated with three or more replicates and the amplified products were pooled to process further for cloning. The amplified products were cloned using pJET1.2 blunt end cloning vector.

### Sequencing and Sequence Analysis

In addition to the standard forward and reverse primers from the pJET1.2 cloning vector, seven additional primers were used for the complete sequence coverage of *Pib* alleles (**Supplementary Table S3**). In addition, a second set of nine primers was designed to cover the sequence gap that might arise from the first round of sequencing, as well as for the re-confirmation of SNPs and/or InDels within the overlap regions. The primers for the isolation of *Pib* alleles and internal primers for sequencing were designed using CLC-genomics workbench.

DNA sequencing was performed using Applied Biosystems Capillary Sequencer 3730. Sequence assembly, consensus development, and alignments were done using CLC Genomics Workbench (version-7.5). The *Pib* sequence from cultivar Engkatek (Accession No. AB013448.1) was used as the reference for sequence comparisons. Multiple sequence alignment was performed to identify the SNPs. Alleles with unique SNPs or InDels were re-amplified (from genomic DNA of respective rice accessions), re-cloned and re-sequenced for SNP/InDel confirmation. For alleles identified in more than four accessions (thereby representing multiple independent amplification and cloning events), no repetitions were made. Sequence polymorphism analyses such as, number of segregation sites and mutations, sliding window analysis of nucleotide diversity and Tajima’s D test were performed using ‘DnaSP, version 5′ (Librado and Rozas, 2009).

### Phylogenetic Analysis

Phylogenetic analysis was performed using full-length nucleotide sequences of the newly identified *Pib* alleles together with reference *Pib_Engkatek*. Phylogenetic analysis was also performed for the NB-ARC-1, NB-ARC-2, and LRR domains separately (**Supplementary Figure S2**). The sequence alignments were performed using MAFFT and the tree was constructed using RAxML on an online server^1^, with default parameters and the bootstrap analysis was performed with 100 replicates.

### Protein Prediction and Analysis

The intron and exon regions were assigned as reported for the reference *Pib* sequence from cv. Engkatek (Wang et al., 1999). The protein sequences of *Pib* alleles were obtained using the EXPASY translation tool. The kinase motifs were assigned as reported for *Pib* (Wang et al., 1999). The CC, NB-ARC and LRR domains were predicted using SMART server (Letunic et al., 2014). The patterns and PTM sites were predicted using ScanProsite server (de Castro et al., 2006). The secondary structure, solvent accessibility, disorders and flexibility regions were predicted and analyzed using PredictProtein server (Yachdav et al., 2014). NetSurfP server was used for sequence-based prediction of the surface accessibility of amino acid residues of *Pib* proteins (Petersen et al., 2009).

#### Accession Numbers

Sequence data from this article can be found in GenBank data libraries with accession numbers, Pib_40286 – KR527222, Pib_16784 – KR527223, Pib_5151 – KR527224, Pib_11147 – KR527225, Pib_29336 – KR527226, Pib_41515 – KR527227, Pib_41341 – KR527228, Pib_32909 – KR527229, Pib_4633 – KR527230, Pib_26038 – KR527231, Pib_24475 – KR527232, Pib_5284 – KR527233, Pib_29436 – KR527234, Pib_13373 – KR527235, Pib_10101 – KR527236, Pib_9829 – KR527237, Pib_14981 – KR527238, Pib_23713 – KR527239, Pib_23761 – KR527240, Pib_23712 – KR527241, Pib_23721 – KR527242, Pib_40432 – KR527243, Pib_16706 – KR527244, Pib_4868 – KR527245, Pib_2476 – KR527246.

## Author Contributions

NB: conceived and designed the experiment, KV: carried out the experiments, CVC: supported the phenotypic screening at IRRI, KV and NB: analyzed the data, KV, WG, and NB: discussed the data, KV and NB: wrote the manuscript, NB and WG: edited the final manuscript. All authors have read the manuscript and agree with its content.

## Conflict of Interest Statement

The authors declare that the research was conducted in the absence of any commercial or financial relationships that could be construed as a potential conflict of interest.

## Footnotes

1 http://www.trex.uqam.ca/index.php?action=raxml&project=trex
