# Supplementary material for: Geographically Distinct and Domain-Specific Sequence Variations in the Alleles of Rice Blast Resistance Gene Pib
Source: Front Plant Sci. 2016 Jun 23;7:915. doi: 10.3389/fpls.2016.00915 (PMC4917536; doi:10.3389/fpls.2016.00915)
Supplement: Supplementary file 3 [file Table_3.PDF]

Supplementary Table 3. List of sequencing primers used for *Pib* sequence coverage

1st set of sequencing primers was the standard set of primers used in sequencing each of the *Pib* alleles. 2nd set of sequencing primers were used only for the samples that showed sequence gaps and/or poor sequence coverage in the overlap regions of sequence results obtained from the 1st set of sequencing primers.

	Primer	Sequence 5' to 3'
1st set of sequencing primers	F1	pJET1.2 forward
	F2	GGGAACAAGTGGTGATAT
	F3	TGAAGTGCGTGGGAGTTA
	R4	GGCCACTGTATGATTGTT
	R5	TTATCTGGTCCCATTCTG
	R6	GGATTCATTTCCAACTCAG
	F7	TAAAGAAGTGCGATGGAC
	R8	ACAGACAGTGATTCCAGC
	R9	pJET1.2 reverse
2nd set of sequencing primers	F1	TGGCCAAGCAGATGAAGGA
	F2	GCCGAATCATAGTGTCAA
	R3	ATATCAAAATTATTTGTG
	R4	CTAGGGTGGTTTTTCCAA
	F5	TGGAAAAACCACCCTAGT
	R6	TGGACTTTGCAAGAGTCA
	F7	GACTCTTGCAAAGTCCAT
	F8	CTTTCGCTTGCTGCGTGA
	F9	CAGGGTGCTCAAGCTTGC
