# Supplementary material for: Geographically Distinct and Domain-Specific Sequence Variations in the Alleles of Rice Blast Resistance Gene Pib
Source: Front Plant Sci. 2016 Jun 23;7:915. doi: 10.3389/fpls.2016.00915 (PMC4917536; doi:10.3389/fpls.2016.00915)
Supplement: Supplementary file 4 [file Image_1.PDF]

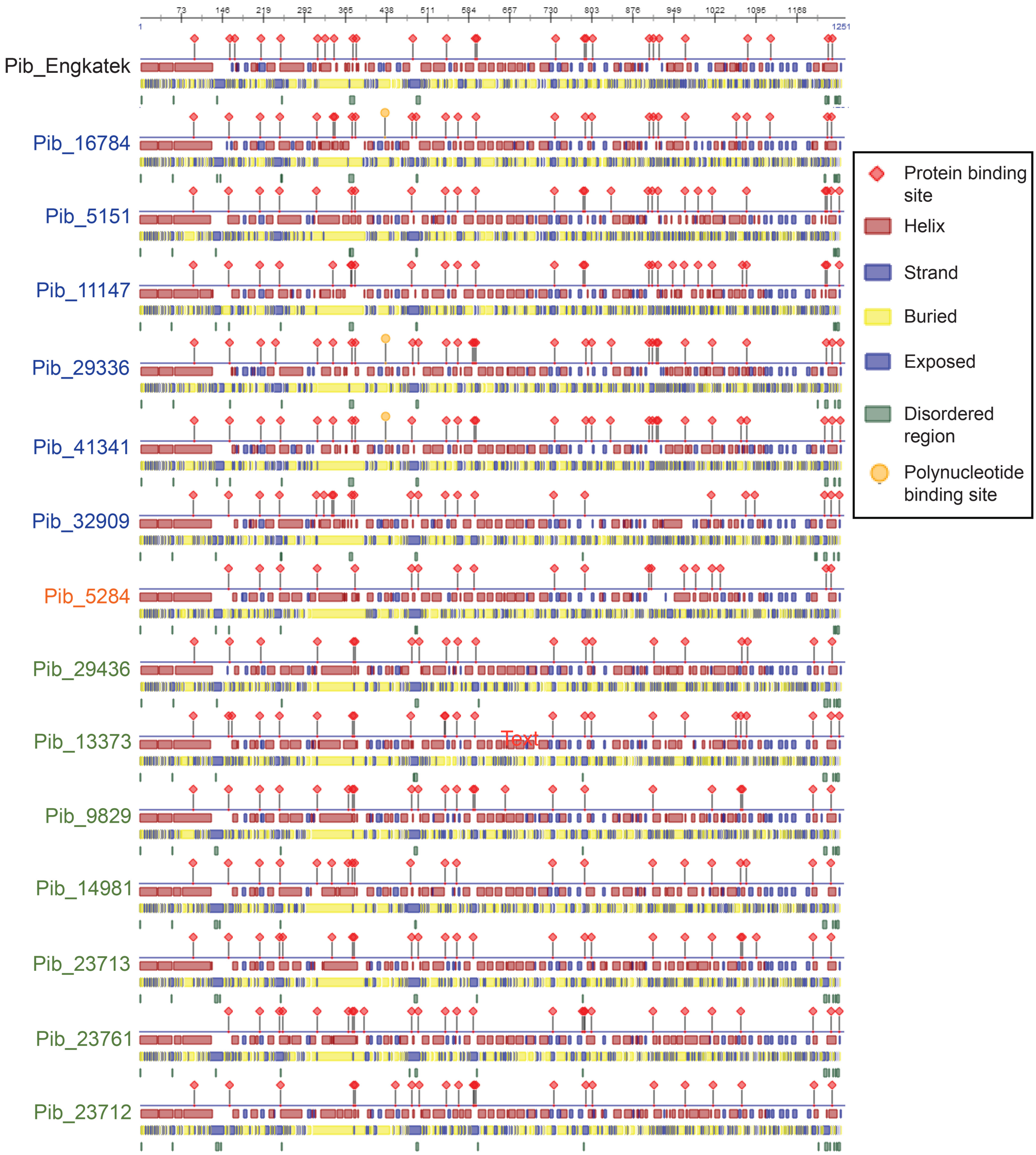


Supplementary Figure S1. Schematic representation of secondary structure elements, exposed/ buried regions, disorder regions and protein binding sites within the Pib proteins.

Protein sequences of alleles possessing complete ORF as that of Pib_Engkatek were subjected for analysis using 'PredictProtein' server for the secondary structure arrangements, solvent accessibility and protein binding predictions. Any two alleles with identical predicted proteins were presented with one of the two protein sequences respectively (see 'Pib proteins have conserved structural domains and post-translational modification sites' part in results section). Proteins labelled in blue, red and green represent Pib category I, IV and II, respectively.
