# Supplementary material for: Geographically Distinct and Domain-Specific Sequence Variations in the Alleles of Rice Blast Resistance Gene Pib
Source: Front Plant Sci. 2016 Jun 23;7:915. doi: 10.3389/fpls.2016.00915 (PMC4917536; doi:10.3389/fpls.2016.00915)
Supplement: Supplementary file 5 [file Image_2.PDF]

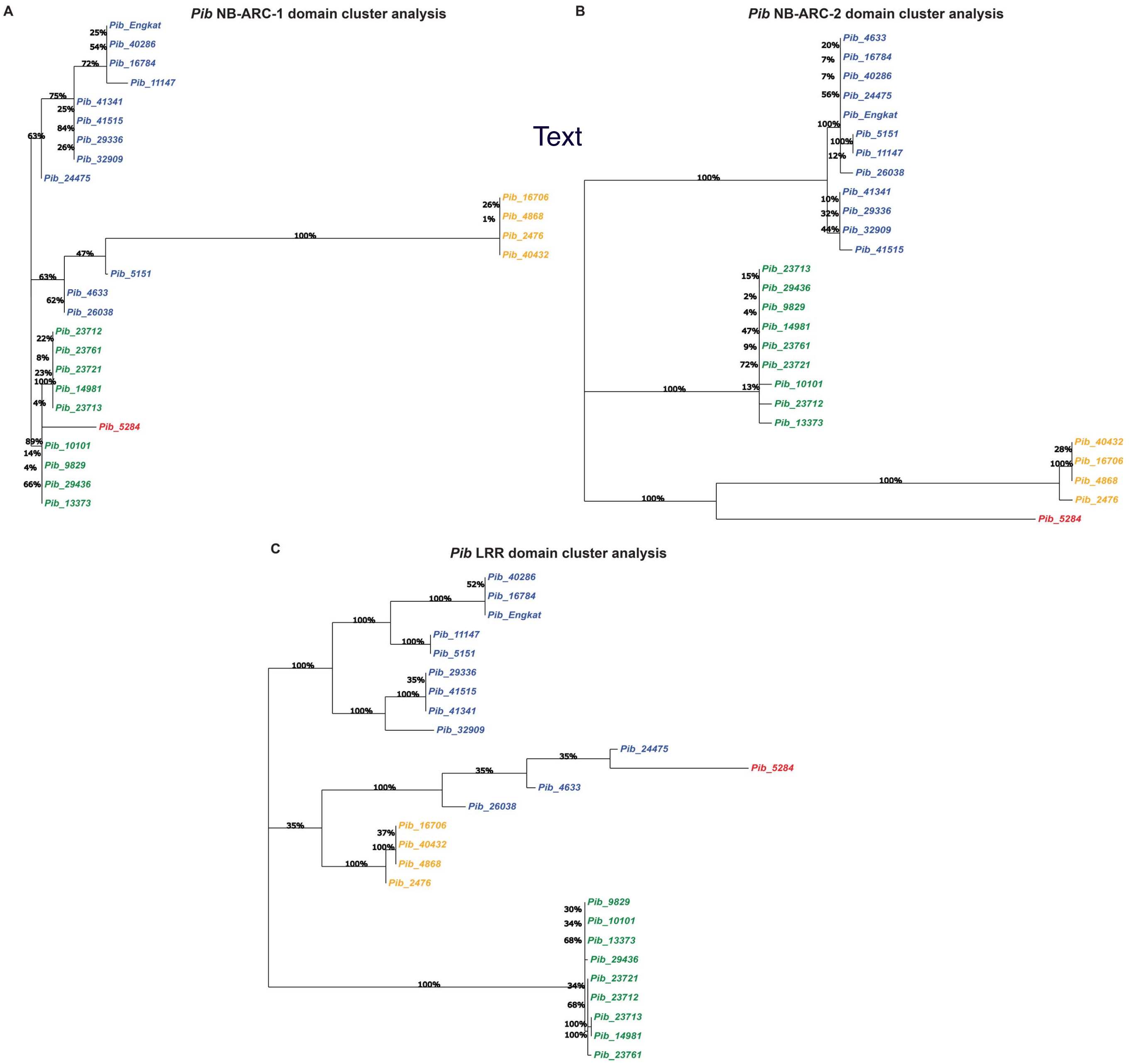

Supplementary Figure S2. Domain specific phylogenetic analysis of the new Pib alleles. Phylogenetic relationship among nucleotide sequences of NB-ARC-1 domain (A), NB-ARC-2 domain (B) and LRR domain (C) from newly identified Pib alleles together with the reference. The alleles labelled in blue, green, orange and red represent category I, II, III, and IV, respectively. The tree was constructed using RAxML program. Bootstrap values (100 replications) are mentioned at the branch nodes.
